# Immunostimulatory oncolytic virotherapy for multiple myeloma targeting 4-1BB and/or CD40

**DOI:** 10.1038/s41417-020-0176-9

**Published:** 2020-05-01

**Authors:** Jessica Wenthe, Sedigheh Naseri, Ann-Charlotte Hellström, Helena Jernberg Wiklund, Emma Eriksson, Angelica Loskog

**Affiliations:** 1 Department of Immunology, Genetics and Pathology, Science for Life Laboratory, Uppsala University, Uppsala, Sweden; 2 Lokon Pharma AB, Uppsala, Sweden

**Keywords:** Myeloma, Immunotherapy, Gene delivery, Applied immunology

## Abstract

Multiple myeloma (MM) is a plasma cell malignancy that is characterized by immune dysregulation. MM is commonly treated with immunomodulating agents, but still remains incurable. Herein, we proposed and evaluated immunostimulatory Lokon oncolytic adenoviruses (LOAd) for MM treatment. LOAd viruses are serotype 5/35 chimera, which enables infection of hematopoietic cells. Oncolysis is restricted to cells with a dysregulated retinoblastoma protein pathway, which is frequently observed in MM. Further, LOAd viruses are armed with human immunostimulatory transgenes: trimerized membrane-bound CD40L (LOAd700, LOAd703) and 4-1BBL (LOAd703). LOAd viruses were assessed in a panel of MM cell lines (ANBL-6, L363, LP-1, OPM-2, RPMI-8226, and U266-84). All cells were sensitive to infection, leading to viral replication and cell killing as analyzed by quantitative PCR and viability assay. Transgene expression was verified post infection with flow cytometry. Cell phenotypes were further altered with a downregulation of markers connected to MM progression (ICAM-1, CD70, CXCL10, CCL2, and sIL-2Rα) and an upregulation of the death receptor Fas. In a co-culture of immune and MM cells, LOAd viruses promoted activation of cytotoxic T cells as seen by higher CD69, CD107a, and IFNγ expression. This was most prominent with LOAd703. In conclusion, LOAd viruses are of interest for MM therapy.

## Introduction

Multiple myeloma (MM) is a plasma cell malignancy and the second most common hematological cancer type with an incidence rate of 5 cases per 100,000 people in the Western world. Even though current treatment options have improved the overall survival to a median of 6 years, MM still remains incurable [1, 2]. Patients with high-risk MM with presence of extramedullary disease (infiltrates in soft tissue or visceral organs) have an especially bad prognosis with no effective treatment options available [3, 4]. Thus, the development of novel therapies is of particular importance for this patient group. Immunotherapies, such as immune checkpoint inhibition and chimeric antigen receptor T cell therapy, have shown great success in a variety of cancers, but these approaches seem to be more challenging in MM [5]. Nevertheless, considering that an important hallmark of MM is immune dysregulation, which facilitates the escape from immune surveillance due to impaired dendritic cell (DC) function and Th1 responses, it is especially intriguing to advance immunotherapeutic approaches for this indication [6]. Specifically, oncolytic virotherapy represents a suitable option as oncolytic viruses are able to enhance the immunogenicity of tumors and induce antitumor immune responses [7]. In addition, MM cells often display overexpression of viral entry receptors (e.g., CD46) and alterations in signaling pathways, which enables viral infection and replication. Previous preclinical and clinical studies in MM have mostly utilized measles virus, reovirus, or vesicular stomatitis virus [8, 9]. Herein, we are investigating oncolytic adenoviruses within the Lokon oncolytic adenovirus (LOAd) platform in human MM models. Commonly used serotype 5 adenoviruses cannot efficiently infect hematopoietic cells due to absence of the coxsackievirus and adenovirus receptor in these cells [10]. LOAd viruses are serotype chimera, meaning that the adenoviral fiber and knob are switched to serotype 35 (Ad5/35) [11]. This retargets the virus to infect CD46+ cells, thereby enabling infection of most cell types, including MM cells. Viral replication is controlled due to a deletion in the viral E1A site (E1AΔ24), thereby restricting lysis to cells with a dysregulated retinoblastoma pathway. Intriguingly, many MM patients display even complete deletions of the retinoblastoma gene, which should drive potent LOAd replication [12–14]. In addition to their oncolytic function, LOAd viruses are further modified to encode for immunostimulatory transgenes in order to enhance antitumor immune responses. Both LOAd700 [11] and LOAd703 [15] encode a designed human trimerized membrane-bound CD40L, but LOAd703 additionally encodes for human wildtype full length 4-1BBL. CD40 stimulating therapy may be of special interest for MM since these cells origin from germinal center B cells and thus have similar features as professional antigen-presenting cells [16]. For instance, stimulation of B cell lymphomas with adenoviruses encoding CD40L has been shown to increase their antigen-presentation capacity and ability to activate antigen-specific cytotoxic T cells [17, 18]. Interestingly, also MM cells have shown to react to CD40L gene therapy in a similar manner. For example, Dotti et al. demonstrated that MM cells infected with an adenovirus carrying CD40L could activate bystander DCs, which in turn could induce T cell responses [19]. Moreover, an oncolytic adenovirus armed with CD40L has been shown to cause enhanced MM cell killing and upregulation of the death receptor Fas [20]. Hence, the aim of this study was to evaluate LOAd viruses armed with CD40L for the treatment of MM by determining the oncolytic and immunostimulatory function of LOAd700 and LOAd703 in preclinical MM models.

## Materials and methods

### Cell culture

The human MM cells lines (ANBL-6, L363, LP-1, OPM-2, RPMI-8226, and U266-84) [21] were provided by Prof Helena Jernberg Wiklund at Uppsala University. All cell lines were maintained in RPMI-1640 supplemented with fetal bovine serum (10%), penicillin (100 U/ml) and streptomycin (100 µg/ml) (all supplements purchased from Thermo Fisher Scientific, Waltham, MA, USA). ANBL-6 medium was further supplemented with 2 ng/ml IL-6 (BioLegend, San Diego, CA, USA). The cell lines were STR typed and tested negative for mycoplasma contamination with MycoAlert^TM^ Mycoplasma Detection Kit (Lonza, Basel, Switzerland) prior experiments. In general, cells were thawed and cultured for two to three weeks (~4–6 passages) before initiation of experiments.

### Virus construction and infection

The construction and expansion of LOAd viruses have been previously described [11]. The viral infectious titers were quantified with a fluorescence-forming unit (FFU) assay [22]. LOAd700 and LOAd703 viruses are both armed with human trimerized membrane-bound CD40L (TMZ-CD40L), whereas LOAd703 is additionally armed with human 4-1BBL. Transgenes are expressed under the control of a CMV promoter. LOAd(−), a virus without transgenes, and a replication-deficient adenovirus 5/35 (Ad5/35 Mock; E1/E3 deleted) were used as controls. A schematic figure of all virus constructs is shown in Supplemental Fig. S1. LOAd viruses were provided by Lokon Pharma AB, Uppsala, Sweden. For virus infection, cells were washed in serum-free medium and pelleted before the respective virus was added (10–200 multiplicity of infection (MOI) = FFU/cell). Cell suspensions were incubated at 37 °C, 5% CO_2_ for 2 h followed by the addition of complete growth medium.

### Cell viability assay

Cells were infected with the respective virus or left uninfected. Two hours after infection, 1 × 10^4^ cells/well were plated in 96-well plates in quadruplicates. Cells were analyzed for their viability 72 h post infection with CellTiter 96 AQueous One Solution MTS reagent (Promega, Fitchburg, WI, USA) according to manufacturer’s instructions.

### DNA isolation and quantitative PCR

Viral DNA was isolated at 2, 24, 48, and 96 h after virus infection using High Pure Viral Nucleic Acid kit (Roche, Basel, Switzerland). Viral replication was assessed using quantitative (q)PCR with primers detecting the adenoviral E4 orf1 transcript (Fw- 5′CATCAGGTTGATTCACATCGG; Rw- 5′GAAGCGCTGTATGTTGTTCTG) [23]. QPCR was performed using iQ SYBR® Green PCR Supermix kit (Bio-Rad, Hercules, CA, USA) and 1:100 diluted viral DNA. The PCR products were continuously measured by the Bio-Rad CFX96 Real-Time detection system for 40 cycles.

### In vivo experiments

Animal experiments were approved by local animal ethics committee (Dnr: 5.8.18-13471/2017) and performed at Uppsala University. Cells from the human MM cell line RPMI8226 mixed 1:1 with Matrigel (Corning, NY, USA) were injected subcutaneously in immunodeficient BALB/c nude mice (5 × 10^6^ cells/mouse, five mice per group). Palpable tumors (8–10 days post tumor injection) were injected 6× (twice per week) with LOAd(−) or LOAd703 (1 × 10^9^ FFU) or phosphate buffered saline (PBS)/saline as negative control. Mice were treated either intratumorally or intravenously depending on the experimental set-up. Tumor growth was monitored by measuring the tumor area (width by height) and mice with tumors over 100 mm^2^ were sacrificed.

### Flow cytometry analysis of immune markers

Cells were infected with the respective virus (100 FFU/cell) or left uninfected. Forty-eight hours after infection, cells were harvested and washed in PBS supplemented with 3 mM EDTA (Thermo Fisher, Waltham, USA) and 0.5% bovine serum albumin (Sigma-Aldrich, Saint Louis, MO, USA). Cells were stained with fluorescent-labeled antibodies targeting CD46 (PE; clone TRA-2-10), CD40L (BV421; clone 24-31), 4-1BBL (PE; clone 5F4), CD40 (APC; clone HB14), 4-1BB (BV421; clone 4B4-1), HLA-ABC (FITC; clone G46-2.6), HLA-DR (APC; clone L243), CD86 (BV421; clone IT2.2), CD70 (PE; clone 113-16), ICAM-1 (FITC; clone HCD54), and Fas (FITC; clone DX2). All antibodies were purchased from BioLegend. After staining, cells were fixed in PBS containing 1% formaldehyde and 3 mM EDTA and analyzed with BD FACS Canto 2 (BD Biosciences, San Jose, CA, USA). The data were evaluated using FlowJo software (FlowJo LLC, Ashland, OR, USA).

### Detection of soluble immune markers

For detection and quantification of soluble immune markers, supernatants from infected (100 ffu/cell) and uninfected cells were collected at 48 h of post infection and analyzed with a MSD multiplex U-PLEX assay (Meso Scale Discovery, Rockville, MD, USA) according to the manufacturer’s protocol.

### Stimulation of peripheral blood mononuclear cells (PBMCs) in multiple myeloma co-cultures

PBMCs were isolated by density centrifugation with Ficoll-Paque (GE Healthcare, Chicago, IL, USA) from healthy donor buffy coats acquired from the blood bank at Uppsala University hospital. PBMCs were cultured alone or co-cultured either with L363 or U266-84 cells in a ratio of 2:1 at a concentration of 1 × 10^6^ cells/mL in 6-well plates (3 × 10^6^ cells in total). After 24 h, cells were infected with the respective virus by direct addition of virus into the respective well (100 FFU/cell). PBMCs cultured alone and one co-culture well were left uninfected. Forty-eight hours of post infection, cells were harvested and cell culture supernatants were taken for IFN-γ detection (Human IFN-γ ELISA development kit, Mabtech AB, Nacka Strand, Sweden). Cells were stained for flow cytometry analysis as described above with the following antibodies purchased from BD Biosciences: CD45 (FITC; clone 2D1), CD16 (PE; clone B73.1), CD56 (PE; clone My31), CD3 (PerCP; clone SK7), CD4 (PE-Cy7, APC-H7; clone SK3), CD8 (APC; SK1), CCR7 (FITC; clone 150503), CD45RA (APC-H7; clone HI100), PD-1 (PE; clone EH12.1), CD69 (FITC; clone L78), CD25 (PE, clone 2A3), CD127 (PE-Cy7; HIL-7R-M21). CD3 (PerCP; clone UCHT1), CD8a (FITC; clone RPA-T8), and CD107a (PE; clone H4A3) were purchased from BioLegend.

### Statistical analysis

GraphPad Prism 8 (GraphPad Software, San Diego, CA, USA) was used for the statistical analysis. For animal experiments, statistically differences in tumor size were analyzed with Kruskal–Wallis test followed by Dunn’s multiple comparison test. Differences in survival were calculated with Log-rank test. For PBMC co-culture experiments, Friedman test followed by Dunn’s multiple comparisons test was used.

## Results

### LOAd platform viruses kill and replicate in human multiple myeloma cell lines

**Fig. 1:**
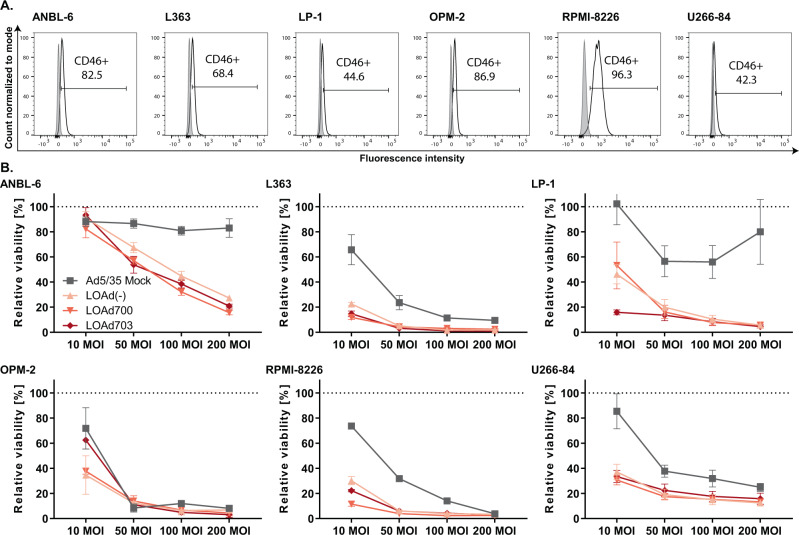
Multiple myeloma cell lines express CD46 and are killed by LOAd viruses. Human multiple myeloma cell lines ANBL-6, L363, LP-1, OPM-2, RPMI-8226, and U266-84 were analyzed for their expression of CD46 by flow cytometry. Gray filled histograms represent matched isotype controls and black lines represent CD46 staining (**a**). All abovementioned cells lines were infected with LOAd viruses or replication-deficient Ad5/35 Mock virus at different virus to cell ratios (10, 20, 50, and 200 MOI). Cell viability was analyzed with MTS viability assay at 72 h post infection and viability is shown as percentage viability of uninfected control cells. *n* = 2; technical replicates = 4; graphs show mean ± SEM.

**Fig. 2:**
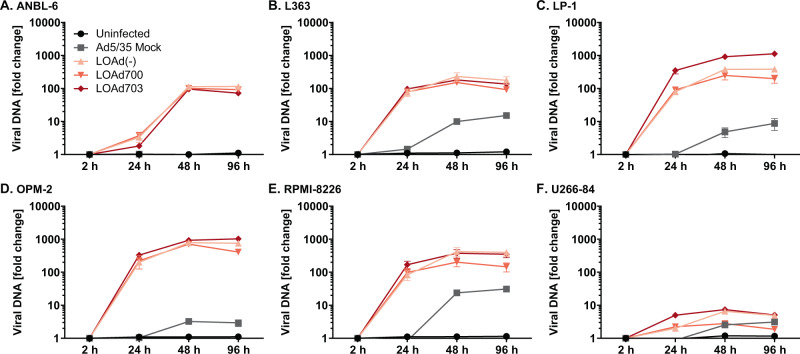
LOAd viruses replicate in multiple myeloma cells. Human multiple myeloma cell lines (ANBL-6 (**a**), L363 (**b**), LP-1 (**c**), OPM-2 (**d**), RPMI-8226 (**e**), and U266-84 (**f**)) were infected with 50 MOI of LOAd viruses or replication-deficient Ad5/35 Mock virus. Viral DNA was isolated at 2, 24, 48, and 96 h post infection and quantified with primers detecting adenoviral DNA. Graphs display the fold change of viral DNA compared to baseline (2 h post infection). *n* = 2; technical replicates = 3; graphs show mean ± SEM.

A panel of six human MM cell lines (ANBL-6, L363, LP-1, OPM-2, RPMI-8226, and U266-84) was selected for the evaluation of LOAd platform viruses for the treatment of MM. LOAd viruses infect cells via the complement regulator CD46, which is frequently overexpressed in many cancer types including MM [9, 24]. All chosen MM cell lines were confirmed to express CD46 with a highest expression level of 96% in RPMI-8226 cells (range in all cell lines: 42–96% CD46+) (Fig. 1a). To determine the oncolytic capacity of LOAd viruses, MM cell lines were infected with LOAd viruses or replication-deficient Ad5/35 Mock virus (E1/E3 deleted) at different virus to cell ratios (10, 20, 50, and 200 MOI). All cell lines were efficiently killed by LOAd viruses 72 h of post infection (Fig. 1b). ANBL-6 was the least sensitive cell line, whereas all other cell lines were also sensitive to Mock virus infection, especially at a higher MOI. OPM-2 appeared especially sensitive as Mock virus killed the cells to a similar extent as LOAd viruses. However, the sensitivity did not seem to correlate with CD46 expression as both ANBL-6 and OPM-2 expressed CD46 to a higher degree (83 and 89%, respectively). To verify that LOAd-infected cells were indeed killed by oncolysis, viral replication was assessed by quantitative PCR. LOAd viruses were shown to replicate in all cell lines as shown by a 100–1000-fold increase of viral DNA over time compared to 2 h of post infection (Fig. 2). In comparison to all other cell lines, viral replication was lowest in U266-84 cells, leading only to a maximum 10-fold increase of viral DNA. Of note, an increase of viral DNA was also detected in Mock-infected cells in 5 out of 6 cell lines, but this increase was much lower (circa 3–30-fold) compared to LOAd-infected cells and may be due to presence of wild type virus (replication competent adenovirus (RCA)) that is often present in adenovirus batches but at low frequencies [25].

### LOAd703 controls tumor growth in a multiple myeloma xenograft model

**Fig. 3:**
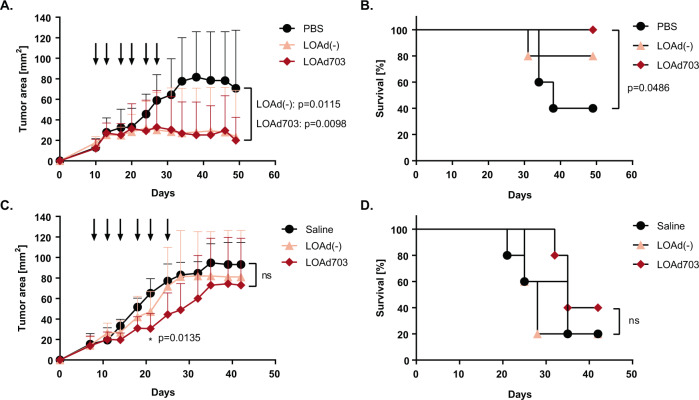
LOAd703 controls tumor growth in vivo. RPMI-8226 cells were subcutaneously injected in immunodeficient BALB/c nude mice (*n* = 5 per group). Palpable tumors were injected 6× with LOAd(−) or LOAd703 (1 × 10^9^ FFU) or PBS/saline as negative control at time-points indicated by arrows in **a** and **c**. Mice were treated either intratumorally (**a**, **b**) or intravenously (**c**, **d**). Mean tumor size + SD is displayed in graphs **a** and **c**. Survival curves are shown in **b** and **d**. Statistically differences in tumor size were analyzed with Kruskal–Wallis test followed by Dunn’s multiple comparisons test. Differences in survival were calculated with Log-rank test.

To investigate the oncolytic function of LOAd viruses in vivo, a xenograft mouse model with subcutaneously injected RPMI-8226 cells was used. Due to fact that adenoviruses are non-enveloped viruses that can be readily neutralized by antibodies, LOAd viruses were so far only evaluated in vivo with intratumoral injections in solid tumors [11, 15]. Nevertheless since MM is a systemic disease, we also wanted to explore intravenous treatments. Note, the effect of the human transgenes cannot be evaluated in a xenograft model since nude mice lack an adaptive immunity and human CD40L cannot activate murine CD40. However, there is a minimal cross reactivity of human 4-1BBL with murine 4-1BB [26]. Hence, to confirm that the oncolytic function of armed LOAd viruses are not impaired in vivo, only LOAd703, which encodes for both CD40L and 41BBL, was used and compared to unarmed LOAd(−). Mice with established tumors (day 8–10 post tumor injection) were treated in total six times (two treatments per week) with PBS/saline, LOAd(−) or LOAd703 either intratumorally (Fig. 3a, b) or intravenously (Fig. 3c, d). Intratumoral injections of both LOAd(−) and LOAd703 were able to control tumor growth (Fig. 3a) compared with PBS control (PBS vs. LOAd(−) *p* = 0.0115, PBS vs. LOAd703 *p* = 0.0098). Moreover, LOAd703-treated mice demonstrated a prolonged survival as compared to the PBS control (*p* = 0.0486) (Fig. 3b). In intravenously LOAd703-treated mice, tumor size was decreased at day 21 compared with saline (*p* = 0.0135), but there was no statistically significant difference in tumor size when analyzing all time-points (Fig. 3c). Likewise, there was no survival benefit in LOAd703-treated mice, but 2/5 mice of this group were alive at the end of experiment (day 42) compared with 1/5 mice in the other two groups (Fig. 3d)

### LOAd infection induces transgene expression in all cell lines

**Fig. 4:**
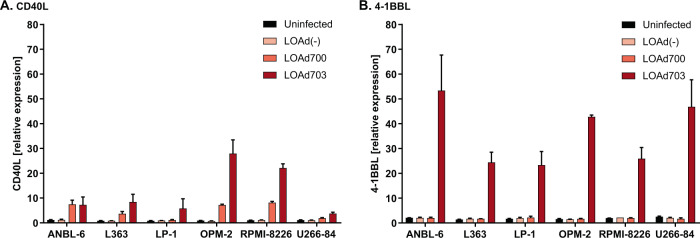
Transgene expression in LOAd-infected multiple myeloma cells. Cells were infected with 100 MOI of LOAd(−), LOAd700, and LOAd703 or left uninfected and cultured for 48 h before cells were harvested for transgene expression analysis by flow cytometry. Bar graphs display the geometric mean fluorescent intensity (MFI) of CD40L (**a**) and 4-1BBL (**b**) divided by the MFI of matched isotype control antibodies (relative expression). *n* = 2; mean ± SEM.

LOAd platform viruses are further modified to encode for human immunostimulatory transgenes, which will be expressed in infected cells independent of virus replication. Both LOAd700 and LOAd703 are modified to encode for a designed trimerized membrane-bound CD40L [11], but LOAd703 additionally encodes 4-1BBL [15]. The control virus LOAd(−) contains no transgenes. The inherent baseline expression of CD40L and 4-1BBL and the corresponding receptors CD40 and 4-1BB in the cell lines is shown in Supplemental Figs. S2–S7. CD40L and was not expressed in any of the uninfected cells, whereas 4-1BBL was expressed to a low extent in all cell lines. CD40 and 4-1BB could not be detected in any of the cells apart from RPMI-8226 cells, which displayed a minor CD40 expression (~6% CD40+). Upon LOAd infection (100 MOI), the respective transgenes were expressed in all MM cell lines as analyzed by flow cytometry and compared to matched isotype controls (Fig. 4). LOAd703 induced higher levels of CD40L than LOAd700 (Fig. 4a), but CD40L expression was overall lower than 4-1BBL expression (Fig. 4b).

### Immune markers on multiple myeloma lines post LOAd infection

**Fig. 5:**
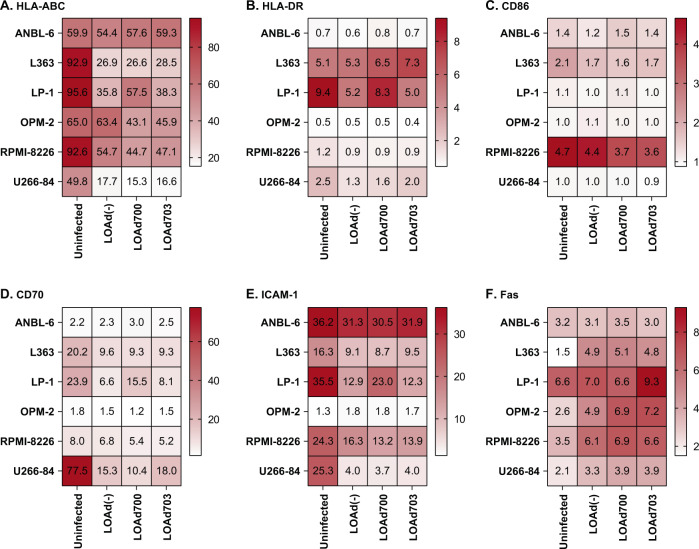
Expression levels of immune markers on LOAd-infected multiple myeloma cells. Cells were infected with 100 MOI of LOAd(−), LOAd700, and LOAd703 or left uninfected, cultured for 48 h and analyzed by flow cytometry for the expression of HLA-ABC (**a**), HLA-DR (**b**), CD86 (**c**), CD70 (**d**), ICAM-1 (**e**), and Fas (**f**). Heat maps display the mean of the geometric mean fluorescent intensity (MFI) of each marker divided by the MFI of matched isotype control antibodies (relative expression). *n* = 2.

MM cells are derived from germinal center B cells and can express markers for antigen presentation and T cell activation. We evaluated if such markers were present and if their expression was altered by LOAd infection. Upregulation of MHC, costimulatory, and adhesion molecules may enable antigen presentation by MM cells and subsequent T cell stimulation, which has been seen by others with induced CD40L expression by adenoviruses [19]. All MM cell lines expressed MHC class I molecules (HLA-ABC) while only three of six (L363, LP-1, and U266-84) displayed MHC class II (HLA-DR) (Fig. 5a, b). Costimulatory and adhesion molecules such as CD86, CD70, and ICAM-1 were present to varying degrees on all lines tested (Fig. 5c–e). The markers were commonly decreased post virus infection, which was more prominent after infection with LOAd700 or LOAd703 that encodes transgenes. Hence, virus replication and expression of transgenes may somehow compete with the cell transcription machinery in these cells due to the oncolysis process, or the transgenes may have a regulatory capacity in MM cells. The same types of markers are upregulated post infection with LOAd viruses in other antigen-presenting cells such as DCs [15]. Interestingly, the apoptosis receptor Fas was upregulated post virus infection in 5 out of 6 cell lines (Fig. 5f).

### LOAd viruses promote CCL3 expression in multiple myeloma lines

**Fig. 6:**
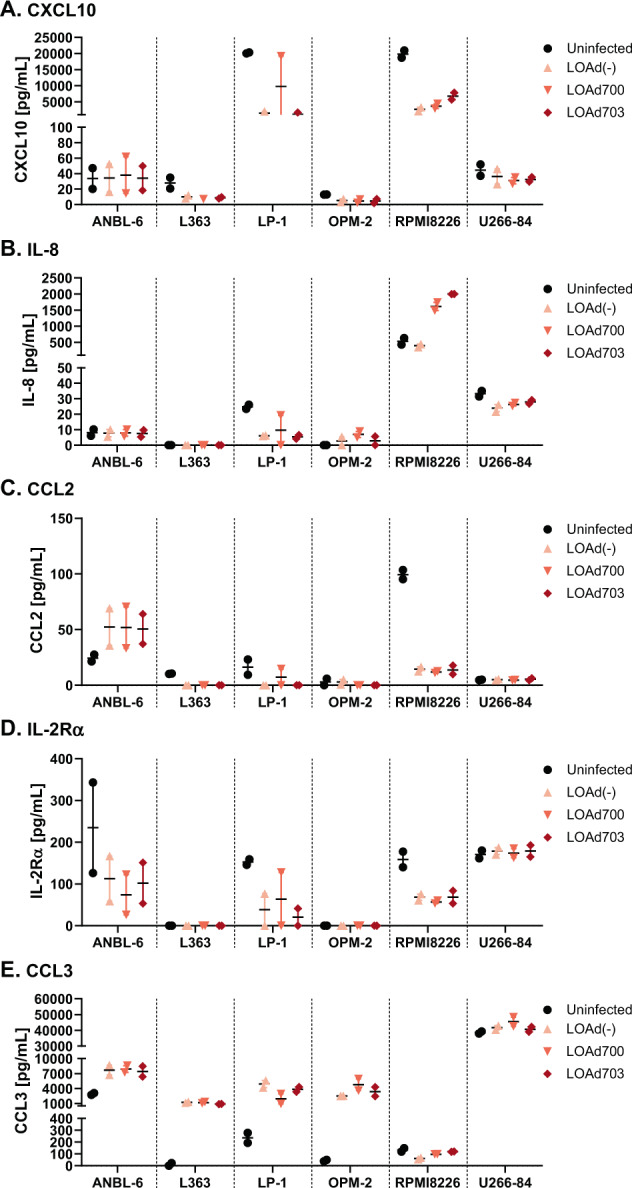
Soluble immune markers in supernatants of LOAd-infected multiple myeloma cells. Cells were infected with 100 MOI of LOAd(−), LOAd700 and LOAd703 or left uninfected. Forty-eight hours post infection, cell culture supernatants were harvested and analyzed by MSD multiplex assay for the presence of CXCL10 (**a**), IL-8 (**b**), CCL2 (**c**), sIL-2Rα (**d**), and CCL3 (**e**). Graphs show the mean concentrations ± SEM of the respective marker in pg/mL. *n* = 2.

The presence of soluble immune markers that are frequently implicated in MM [27–30] was analyzed pre-LOAd and post-LOAd infection of MM cells using MSD multiplex assay. The analytes IFNγ, IL-1RA, CCL22, CCL17, and TNFα were undetectable in ANBL-6, L363, and OPM-2 and only present at low amount and to a varying degree in the other cell lines (Supplemental Fig. S8). However, most lines expressed CXCL10, IL-8, CCL2, sIL-2Rα, and CCL3 (Fig. 6). Overall, these soluble markers were decreased post infection that can be due to the rapid onset of the oncolysis process, which ultimately leads to less time to release these markers. However, CCL3 was increased by LOAd infection, except in RPMI-8226 cells (Fig. 6e). Contrarily, RPMI-8226 cells had increased levels of IL-8 post infection of LOAd700 and LOAd703 (Fig. 6b).

### Transgene expressing LOAd viruses enhance T cell activation in multiple myeloma co-cultures

**Fig. 7:**
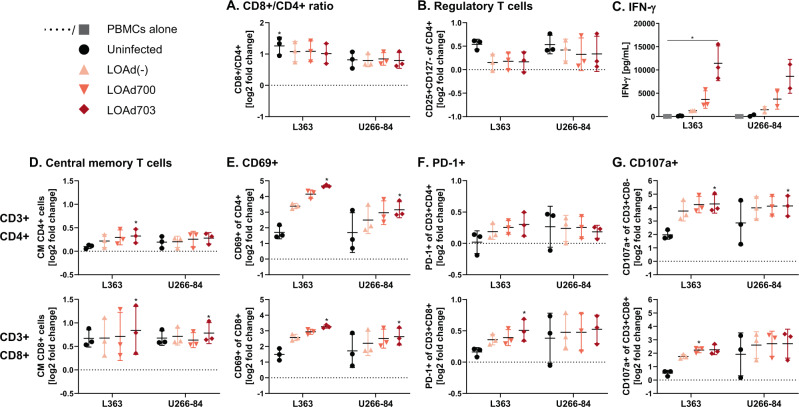
LOAd viruses activate T cells in a co-culture of PBMC and multiple myeloma cells. Peripheral blood mononuclear cells (PBMCs) derived from healthy donor buffy coats (*n* = 3) were co-cultured with L363 or U-266-84 cells (2:1 ratio; 1 × 10^6^ cells/mL) and 24 h later infected with 100 MOI of LOAd(−), LOAd700 and LOAd703 or left uninfected. Forty-eight hours post infection, cells were analyzed by flow cytometry for T cell markers and culture supernatants were analyzed for IFN-γ by ELISA. Flow cytometry results (**a**, **b**, **d**–**g**) are displayed as mean log2 fold change of percentage positive cells to PBMCs cultured alone (dotted line) ± SD. **c** Mean IFN-γ concentrations (pg/mL) ± SD. Statistically differences were analyzed with Friedman test followed by Dunn’s multiple comparisons test (*p < 0.05).

The immunological assessment of the armed LOAd viruses for MM treatment would ideally require an immunocompetent syngeneic mouse model. However, as the receptor for viral entry, CD46, is not expressed in murine cells and adenoviruses cannot replicate in murine cells [31], it is impossible to evaluate this therapy fully in these models, even if a virus construct with murine transgenes would be used. Hence, we examined in an in vitro model if LOAd viruses can activate immune cells conditioned in the presence of MM cells. For this, a co-culture of MM cells together with healthy donor PBMCs was set-up and 24 h later infected with LOAd viruses. We tested this experimental set-up with two of the six MM cell lines, L363 and U266-84, in order to examine if there is any difference in the magnitude of immune cell activation dependent on the virus replication and subsequent phenotype changes in these cell lines. L363 cells allowed efficient virus replication (average across all cell lines), whereas the replication was lowest in U266-84 cells. In addition, L363 cells expressed the T cell costimulatory molecule CD86 and upregulated HLA-DR expression upon infection with the armed viruses. In contrast, U266-84 cells did not express CD86 and displayed the greatest reductions in CD70 and ICAM-1 upon LOAd infection. The co-cultures were analyzed with flow cytometry analysis 48 h post infection. As the majority of cells in culture were CD3+, the T cell compartment was investigated further. The gating strategy for flow cytometry analysis is shown in Supplemental Fig. S9. In general, co-culture with either MM cell line altered the CD8+/CD4+ ratio towards CD8+ T cells (Fig. 7a). However, presence of MM cells also expanded CD25+ CD127− regulatory T cells (Tregs), but these cells were reduced in LOAd-infected cultures (Fig. 7b). CD8+ and CD4+ T cells were further analyzed for phenotype changes based on CD45RA and CCR7 expression. Interestingly, CD45RA-CCR7+ central memory T cells were increased in both subsets in particular in cultures with LOAd703-infected cells (Fig. 7d). The activation status of T cells was assessed by checking the expression of activation markers CD69, PD-1, and degranulation marker CD107a (Fig. 7e–g). These markers were overall upregulated in the presence of LOAd virus infection with the highest level noted in the LOAd703 group. Likewise, cells activated by LOAd703 released IFN-γ to a significantly greater extent than cells infected with LOAd700 or LOAd(−), or uninfected controls (Fig. 7c). Overall, the T cell activation profile was comparable between the two cell lines used. Nevertheless, CD69 and IFNγ expression levels were slightly lower in co-cultures with U266-84 cells, which is in agreement with the lower levels of co-stimulatory molecules and reduced viral replication in this cell line.

## Discussion

Even though MM remains a largely incurable disease, treatment regimens including immunomodulatory drugs, such as lenalidomide, have clearly improved the outcome of patients [32]. Lenalidomide not only causes direct cytotoxicity in MM, but also has pleiotropic effects on the immune compartment [33]. Therapies targeting the immune system may be of particular interested in MM as patients with MM display notably impaired immune cell functions as seen by their increased susceptibility to infections [34]. Thus, this study aimed to determine if immunostimulatory oncolytic virotherapy within the LOAd platform may be a suitable treatment option for MM. Given that LOAd viruses are serotype Ad5/35 chimera, their serotype 35 fiber makes it possible to infect cells of hematopoietic origin via CD46. In fact, malignant plasma cells have been found to express CD46 to a much higher degree than normal hematopoietic cells [9]. This increased CD46 expression in MM cells has been shown to be regulated by p53 deficiency [35]. Serotype 35 adenoviruses have been reported to bind to MM cells up to 100 times better than other serotypes, whereas serotype 5 adenoviruses have showed more potent replication and MM cell killing [36]. Therefore, the chimeric serotype of LOAd viruses should facilitate both features in MM.

Indeed, all MM cell lines tested were very sensitive to viral infection at even low virus to cell ratio and efficiently killed. Also the replication-deficient Ad5/35 Mock virus could seemingly alter the cell metabolism of infected cells, which is the readout of the MTS viability assay used in the killing studies. Nevertheless, LOAd-infected MM cells were killed via oncolysis as seen by an up to 1000 fold increase of adenoviral DNA post infection. The minor replication of Mock virus observed in the MM cell lines may have been due to the potential presence of low numbers of wild type virus (RCA) in the Mock virus batch [25]. LOAd viruses were also able to control tumor growth through oncolysis in an in vivo xenograft model when injected intratumorally. There was no difference in the in vivo oncolytic function between LOAd(−) and LOAd703 expressing CD40L and 4-1BBL. Fernandes et al. observed enhanced tumor reductions in a RPMI-8226 xenograft model with a CD40L expressing oncolytic adenovirus [20]. However, the RPMI-8226 cells used herein had a reduced CD40 expression (6%) compared to their study (50%). Therefore, the lack of CD40/CD40L signaling inducing apoptosis [37] in the cells may be the reason for these contrasting results. Intravenous injections failed to control tumor growth, which is likely due to reduced delivery of virus particles to the actual tumor site upon intravenous injection. In humans, intravenous injections may be problematic due to the fact that adenoviruses are non-enveloped viruses, which can be readily cleared from the blood through opsonization by antibodies and complement [38] before reaching the tumor site. Thus, intratumoral delivery of LOAd viruses is favorable. Regardless, LOAd703 seemed to have a slightly better effect initially in the intravenous setting. Human 4-1BBL expressed by LOAd703 can slightly cross react with murine 4-1BB, whereas human CD40L cannot cross react with murine CD40 [26]. Therefore, the initial reduction in tumor growth by LOAd703 compared with LOAd(−) may be explained by 4-1BBL-mediated activation of innate immune cells, which are present in the nude mice used. Unfortunately, there is currently no suitable model available to analyze both the oncolytic and immunostimulatory function of LOAd viruses in vivo. Therefore, all investigations in regard to the effect of the transgenes were evaluated in vitro.

Next, we explored the transgenes effects on the MM cells as well as co-cultured immune cells. CD40L expression was observed upon infection with LOAd700 and LOAd703 and 4-1BBL expression was highly induced in all LOAd703-infected cells. Since malignant plasma cells express similar molecules as professional antigen-presenting cells [16], we hypothesized that infection with CD40L expressing LOAd viruses could further alter the phenotype of MM cells as seen by others with CD40 stimulating therapy [19, 20, 39]. For example, in a study by Bashey et al., CD40 expressing MM cells infected with a CD40L encoding adenovirus upregulated the expression of HLA-DR, ICAM-1, and CD70 and could stimulate T cell proliferation in allogeneic mixed lymphocyte reactions [39]. The MM cell lines used in this study were found to be CD40 negative, apart from a weak CD40 expression in RPMI-8226 cells, and infection with LOAd700 or LOAd703 did not upregulate the abovementioned markers. In fact, MHC molecules as well as CD70 and ICAM-1 were rather downregulated upon LOAd infection in general, indicating that transcription levels may in some way be decreased due to the viral replication process. Downregulation of ICAM-1 could also be beneficial as adhesion molecules are known to mediate the interaction between MM and stromal cells, thereby facilitating MM growth and survival [40]. Also, high ICAM-1 expression has been connected to advanced disease and resistance to chemotherapy [41, 42]. Similarly, high CD70 expression has been detected on MM cells and been proposed as a therapeutic target [43]. In concert with these downregulations, an increase in the expression of the apoptosis receptor Fas was observed, which was most apparent in LOAd700 and LOAd703 infected cells. Hence, this may facilitate Fas/FasL mediated killing of the MM cells by T cells. In agreement, Fernandes et al. also observed an upregulation of Fas in MM cells infected with an oncolytic adenovirus encoding for CD40L [20].

Most investigated soluble immune markers released by MM cells were either stable or primarily reduced upon LOAd infection. These included CXCL10, IL-8, CCL2, and IL-2Rα. CXCL10 is generally seen as a pro-inflammatory chemokine, which is essential for the recruitment of Th1 lymphocytes to the tumor site [44]. However, its receptor CXCR3 is also overexpressed on MM cells and signaling through the receptor has been shown to promote chemotaxis and secretion of matrix metalloproteinases, which drive tumor invasion [45]. CCL2 can be induced by the MM growth factor IL-6 [46] and high CCL2 plasma levels in MM patients have been associated with angiogenesis and advanced disease [47]. Likewise, MM patients display increased levels of soluble IL-2Rα, which have been found to correlate negatively with progression-free survival [30]. CCL3 is another chemokine, which has been associated with poor prognosis and bone disease in MM patients [48]. But in contrast to the other factors, CCL3 was actually increased in the majority of cell lines post LOAd infection. Nevertheless, CCL3 could potentially also have a positive role in facilitating antitumor immune responses by recruiting DCs to the tumor site [49, 50], especially in combination with the pro-inflammatory environment commonly established with oncolytic viruses [7]. Of note, we have previously shown that both LOAd700 and LOAd703 are able to potently activate immature DCs [11, 15], which may be a crucial feature for establishing immune responses against MM cells as impaired DC functions have been frequently described in MM patients [51].

The immune dysregulation observed in MM patients also encompasses alterations in the T cell compartment [34], including shifts in the Th1/Th2 balance [52] and increased Treg levels [53]. Hence, we investigated the effect of LOAd viruses on different T cell subsets and their activation status in MM co-cultures. The CD8+/CD4+ T cell ratio was increased upon co-culture with either MM cell line and was left unchanged upon addition of LOAd viruses. As expected, Tregs were likewise increased in tumor co-cultures, but the levels were reduced again when cells were infected with LOAd viruses. When analyzing the T cell phenotype, a significant increase in central memory T cells was noted in samples infected with LOAd703. Higher levels of memory T cells is generally associated with a favorable outcome across cancer types [54] and lenalidomide has also been suggested to partly exert its effect through polarizing T cells into memory T cells [55]. Zelle-Rieser et al. analyzed the exhaustion profile and functionality of T cells from MM patients and they demonstrated that T cells at the tumor site were functionally impaired as they failed to both increase the degranulation marker CD107a and to produce IFNγ in response to stimulation [56]. Upon infection with LOAd viruses, both CD4+ and CD8+ T cells were able to highly upregulate CD107a as well as the activation marker CD69 in co-culture with MM cells. The highest upregulation was typically observed with LOAd703, which is encoding both CD40L and 4-1BBL. In addition, LOAd703 induced significantly higher IFNγ levels than all other conditions, which is likely due to the additional stimulation of T cells through 4-1BBL/4-1BB signaling [57].

Overall, oncolytic virotherapy within the LOAd platform was shown to be a suitable candidate for the treatment of MM. Typical features of MM cells, such as overexpression of CD46 [9] and a dysregulated retinoblastoma pathway [12–14], enabled LOAd viruses to infect and kill a variety of MM cell lines, both in vitro and in vivo. Treatment in vivo was most effective when injected intratumorally. Even though MM is a systemic disease and intravenous injections may be desired, the patient group that has the biggest unmet clinical need also presents with extramedullary tumor sites, which may serve as a target lesion for LOAd injection. Interestingly, LOAd infection in MM cells resulted in the downregulation of markers associated with MM growth and survival and increased their immunogenicity by upregulating Fas. Most strikingly, LOAd viruses, in particular LOAd703, were able to activate and polarize T cells towards a Th1 response in a co-culture with MM cells. These results warrant further investigations of LOAd703 in MM and it would be of interest to explore the effect of the transgenes on the tumor stroma.

## Supplementary information

Supplemental Figure
