# Supplementary material for: Immunostimulatory oncolytic virotherapy for multiple myeloma targeting 4-1BB and/or CD40
Source: Cancer Gene Ther. 2020 May 1;27(12):948–59. doi: 10.1038/s41417-020-0176-9 (PMC7725669; doi:10.1038/s41417-020-0176-9)
Supplement: Supplementary file 1 — Supplemental Figure [file 41417_2020_176_MOESM1_ESM.pdf]

Supplemental Figure S1: Schematic figure of virus constructs

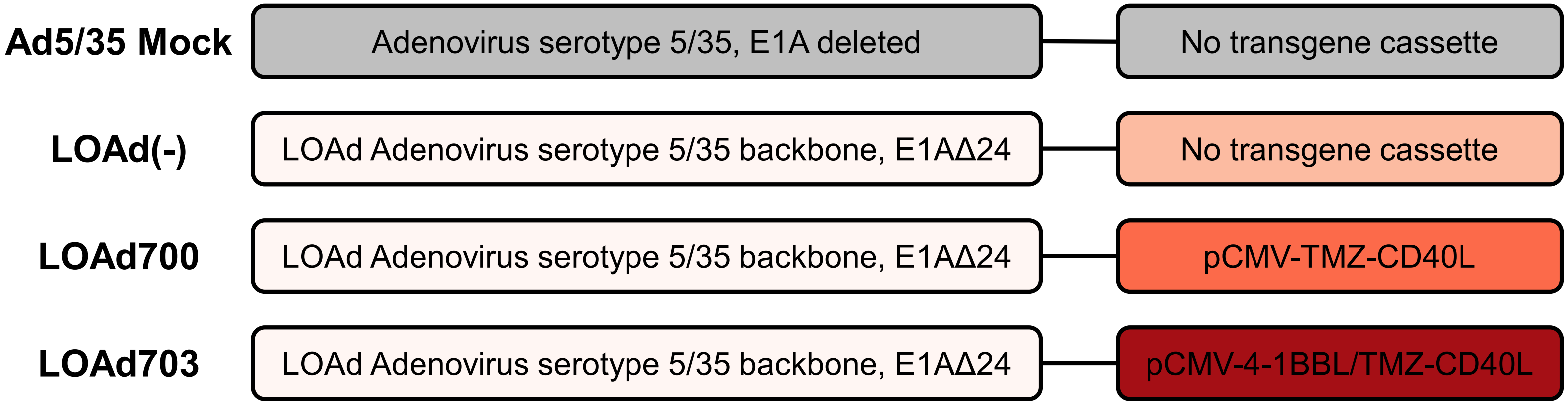

Figure S1: Schematic figure of virus constructs. Ad5/35 Mock is a replication deficient adenovirus serotype 5/35, which has a deletion of E1A. All LOAd viruses are based on the LOAd adenovirus serotype 5/35 backbone with a delta 24 deletion in E1A. For LOAd700 and LOAd703, a transgene cassette driven by a CMV promoter was added downstream. LOAd700 encodes for TMZ-CD40L and LOAd703 encodes for 4-1BBL and TMZ-CD40L. Ad5/35 Mock and LOAd(-) do not contain a transgene cassette.

Supplemental Figure S2: ANBL-6 baseline expression

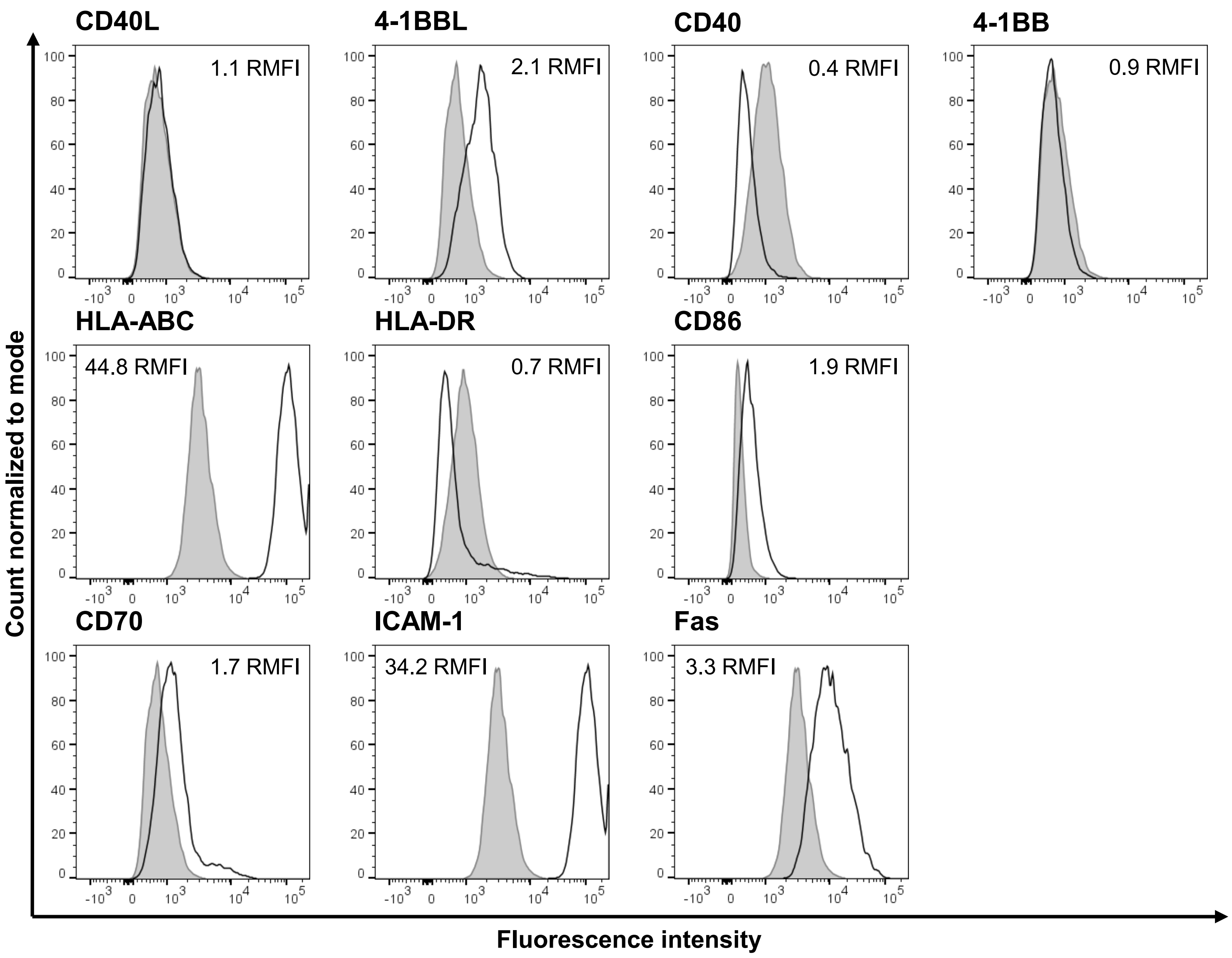

Figure S2: Histogram overlays display the baseline expression (uninfected) of investigated markers in ANBL-6 cells. Grey filled histograms represent matched isotype controls and black lines represent the staining of the respective marker. RMFI (relative mean fluorescence intensity) values show the relative expression of each marker compared to the matched isotype control.

Supplemental Figure S3: L363 baseline expression

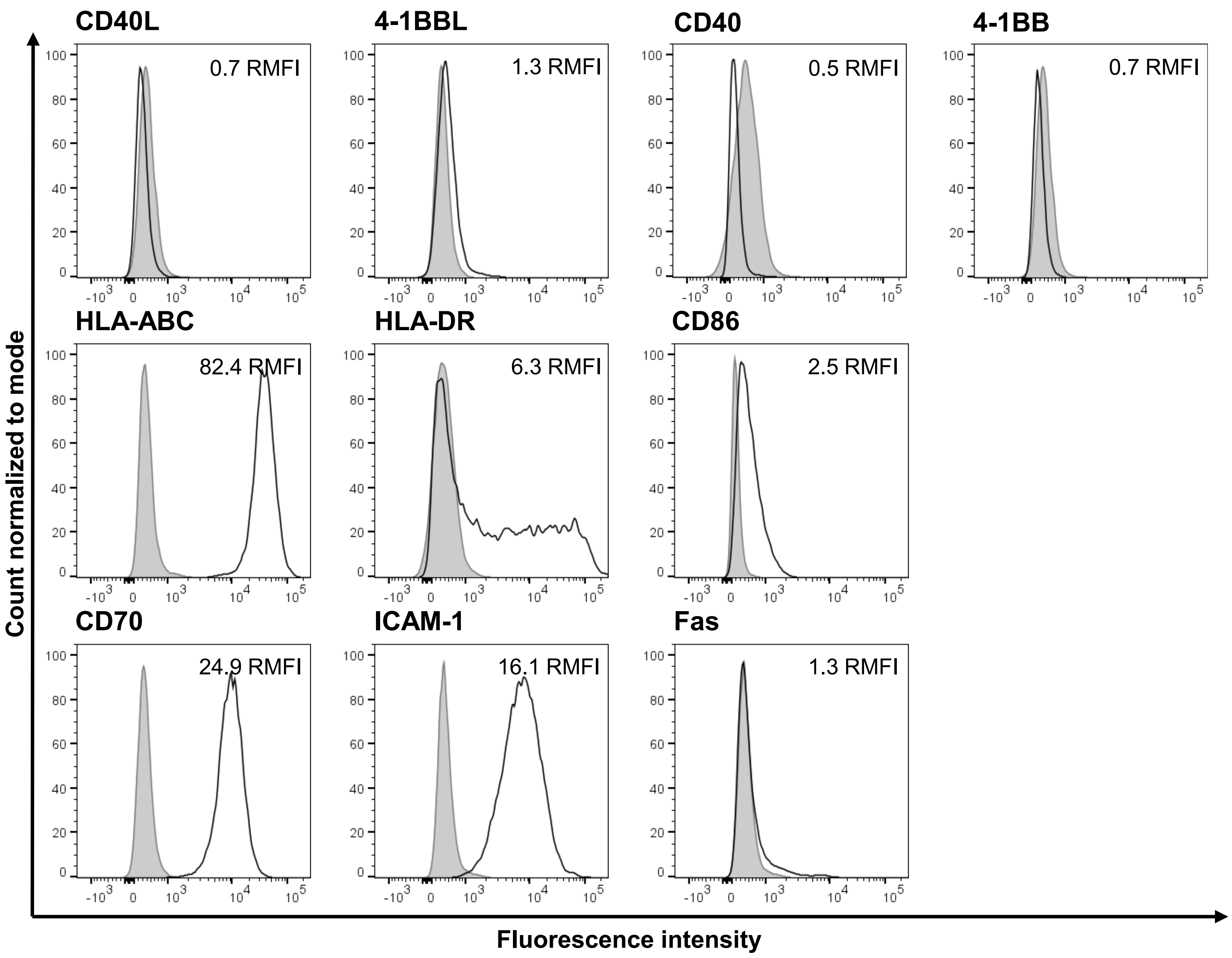

Figure S3: Histogram overlays display the baseline expression (uninfected) of investigated markers in L363 cells. Grey filled histograms represent matched isotype controls and black lines represent the staining of the respective marker. RMFI (relative mean fluorescence intensity) values show the relative expression of each marker compared to the matched isotype control.

Supplemental Figure S4: LP-1 baseline expression

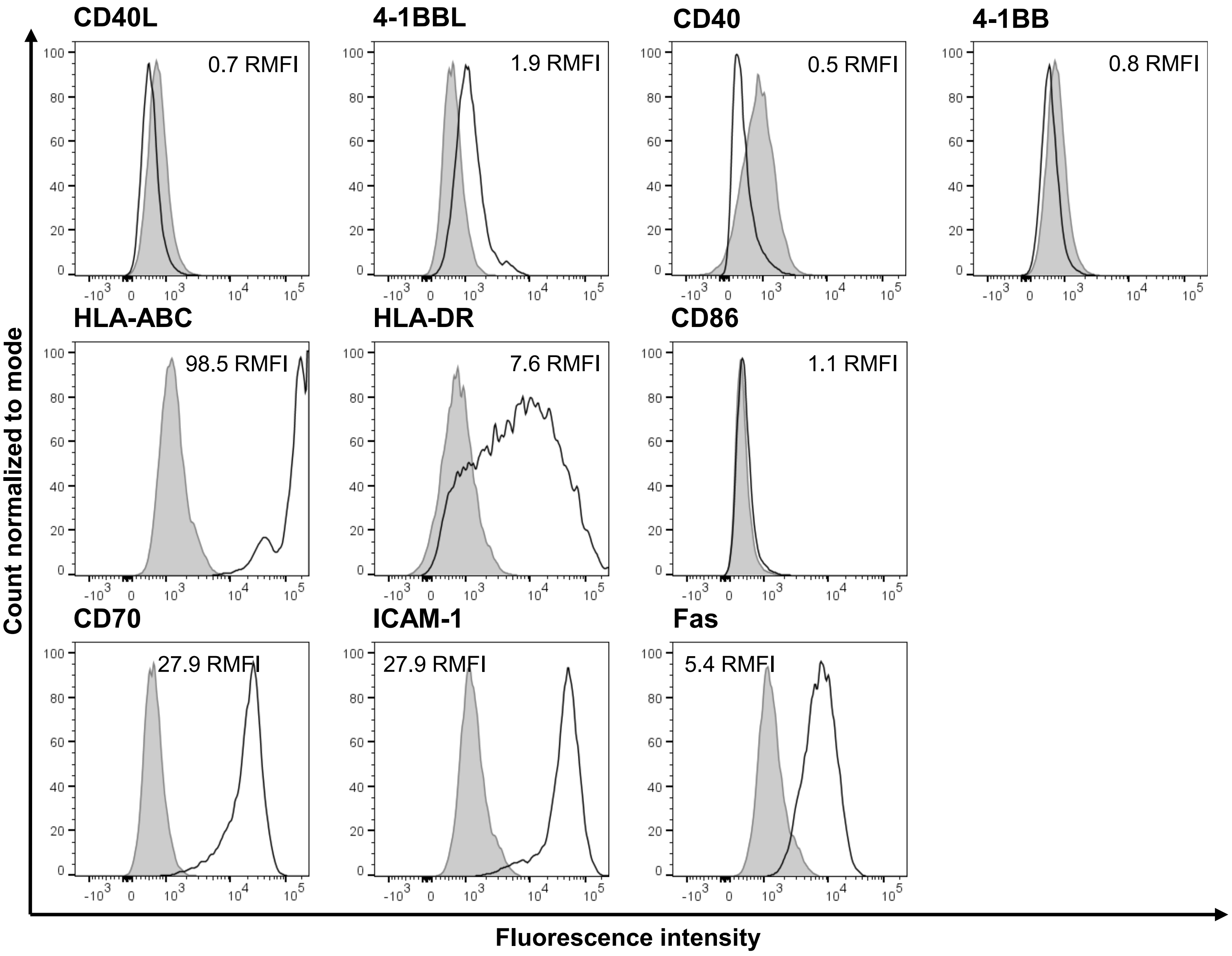

Figure S4: Histogram overlays display the baseline expression (uninfected) of investigated markers in LP-1 cells. Grey filled histograms represent matched isotype controls and black lines represent the staining of the respective marker. RMFI (relative mean fluorescence intensity) values show the relative expression of each marker compared to the matched isotype control.

Supplemental Figure S5: OPM-2 baseline expression

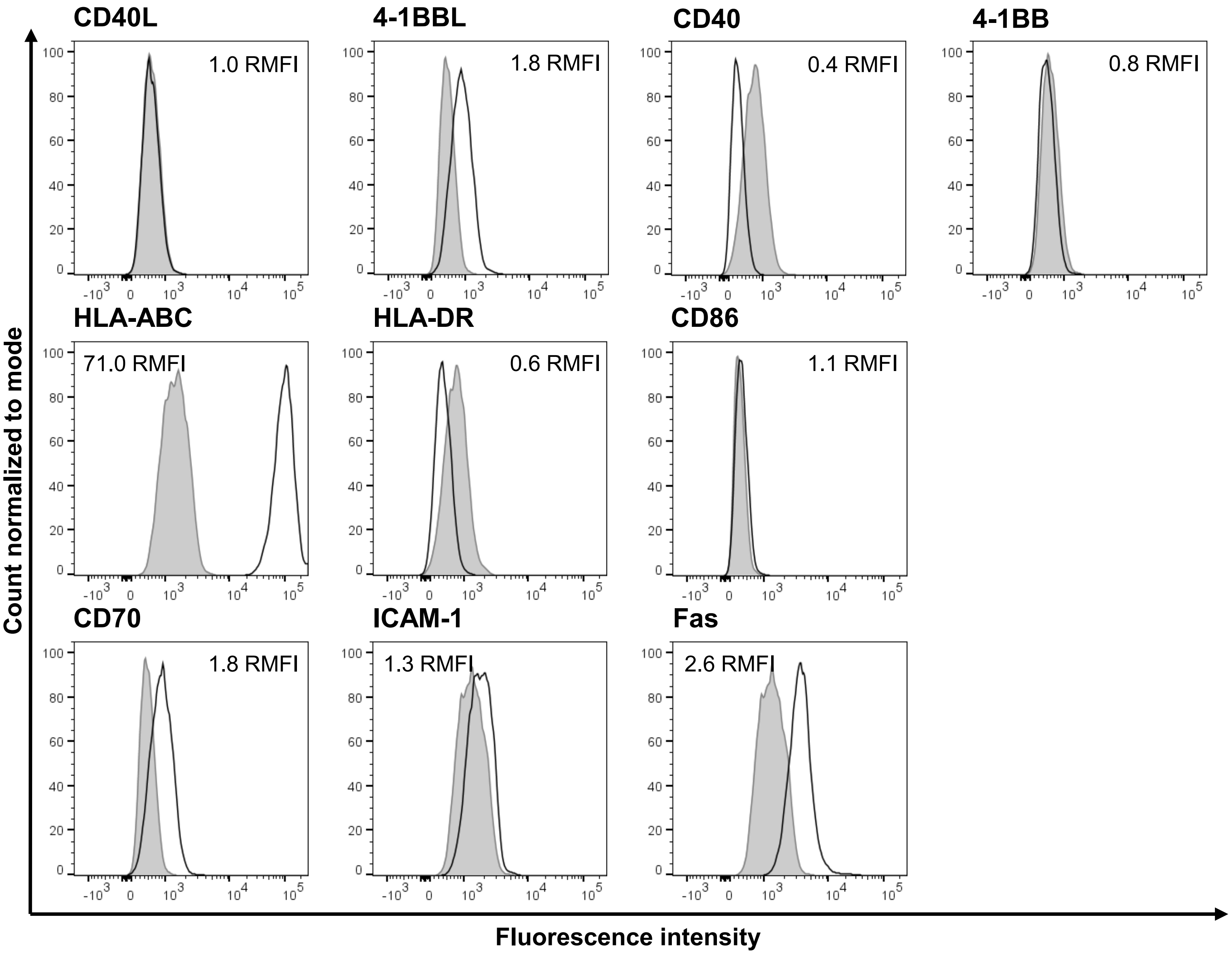

Figure S5: Histogram overlays display the baseline expression (uninfected) of investigated markers in OPM-2 cells. Grey filled histograms represent matched isotype controls and black lines represent the staining of the respective marker. RMFI (relative mean fluorescence intensity) values show the relative expression of each marker compared to the matched isotype control.

Supplemental Figure S6: RPMI-8226 baseline expression

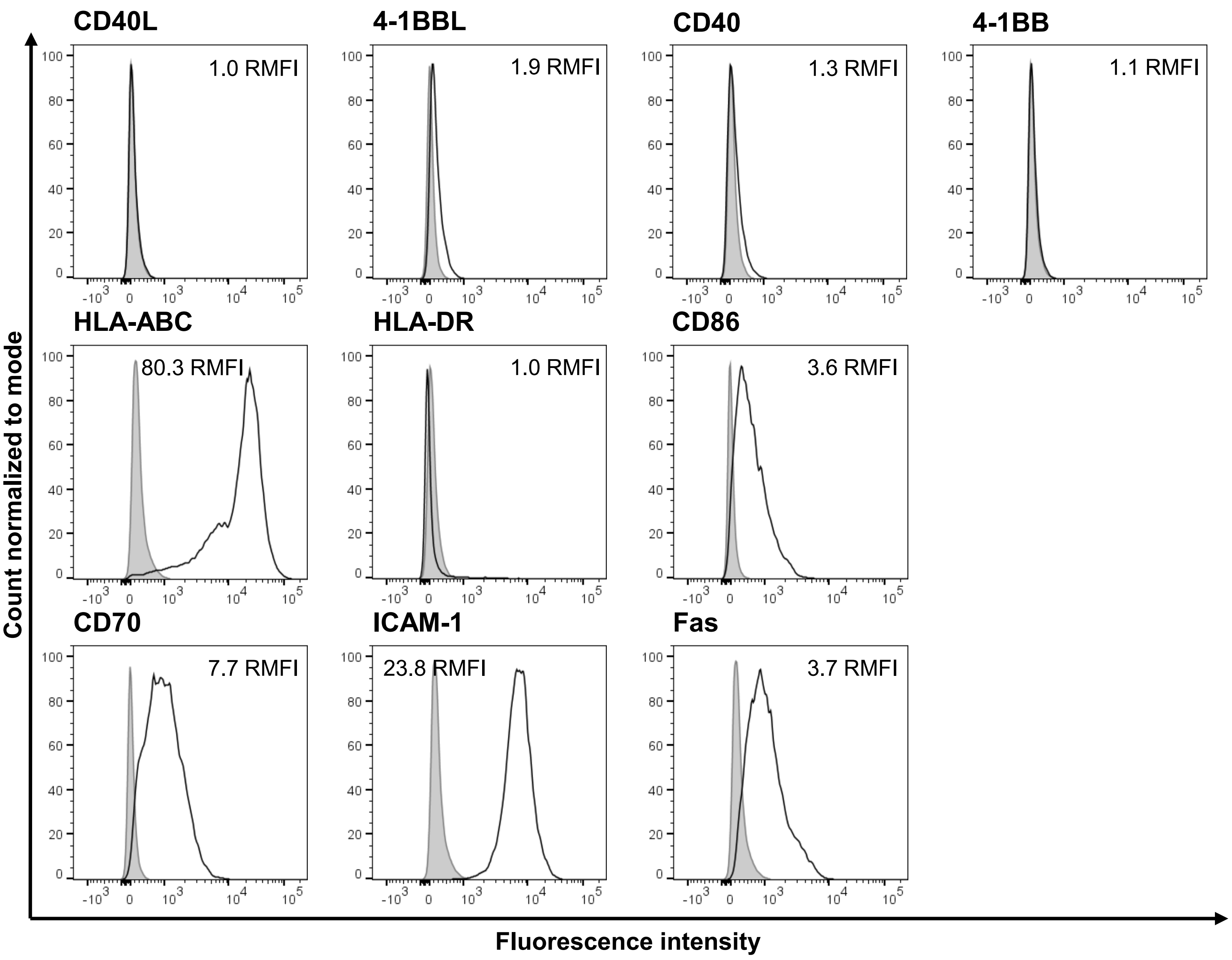

Figure S6: Histogram overlays display the baseline expression (uninfected) of investigated markers in RPMI-8226 cells. Grey filled histograms represent matched isotype controls and black lines represent the staining of the respective marker. RMFI (relative mean fluorescence intensity) values show the relative expression of each marker compared to the matched isotype control.

Supplemental Figure S7: U266-84 baseline expression

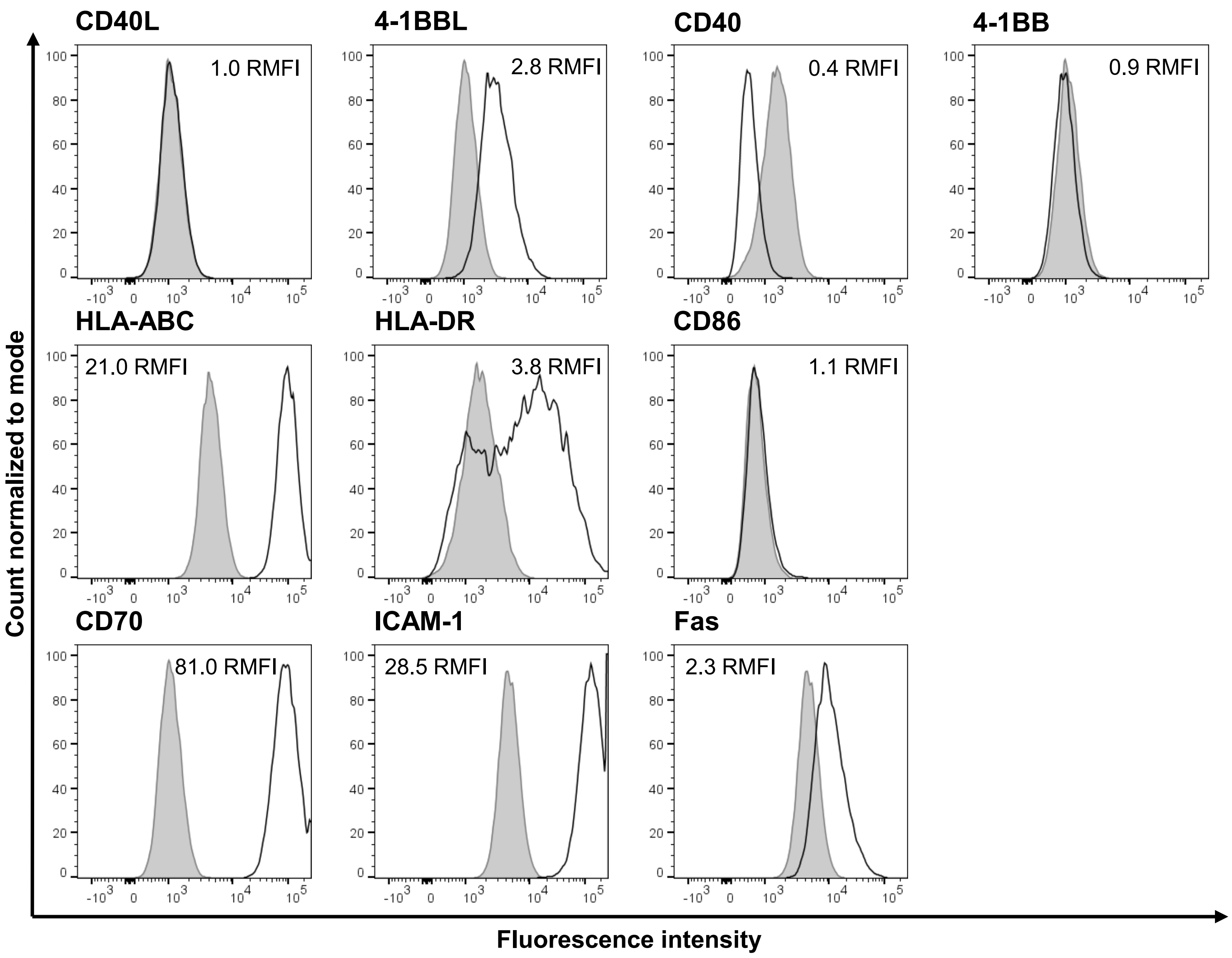

Figure S7: Histogram overlays display the baseline expression (uninfected) of investigated markers in U266-84 cells. Grey filled histograms represent matched isotype controls and black lines represent the staining of the respective marker. RMFI (relative mean fluorescence intensity) values show the relative expression of each marker compared to the matched isotype control.

Supplemental Figure S8: Soluble immune markers in supernatants of LOAd-infected multiple myeloma cells

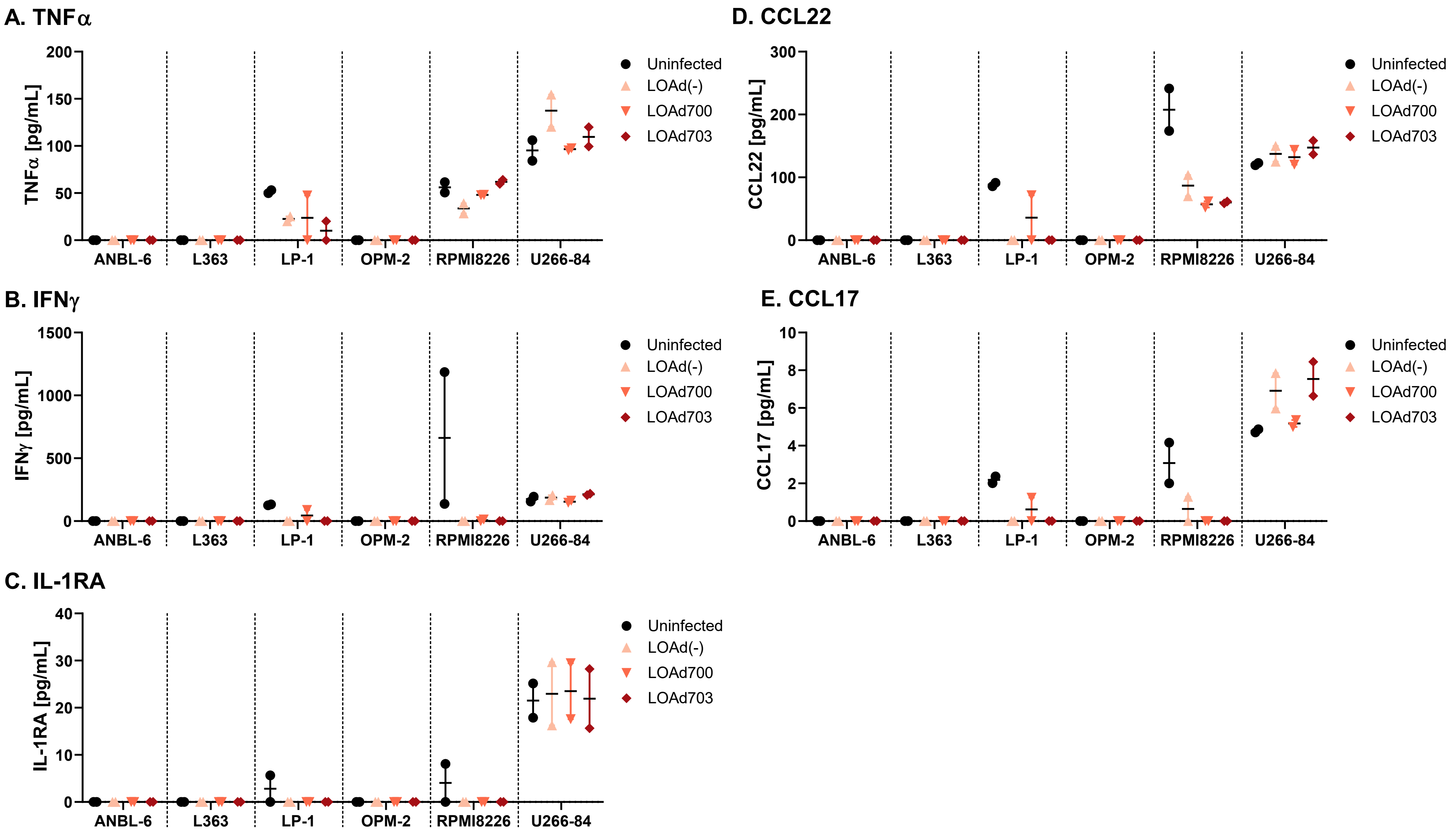

Figure S8: Cells were infected with 100 MOI of LOAd(-), LOAd700 and LOAd703 or left uninfected. 48 hours post infection, cell culture supernatants were harvested and analyzed by MSD multiplex assay for the presence of TNFα (A), IFNγ (B), IL-1RA (C), CCL22 (D), CCL17 (E). Graphs show the mean concentrations ± SEM of the respective marker in pg/mL. n=2

Supplemental Figure S9: PBMCs + L363 + LOAd703

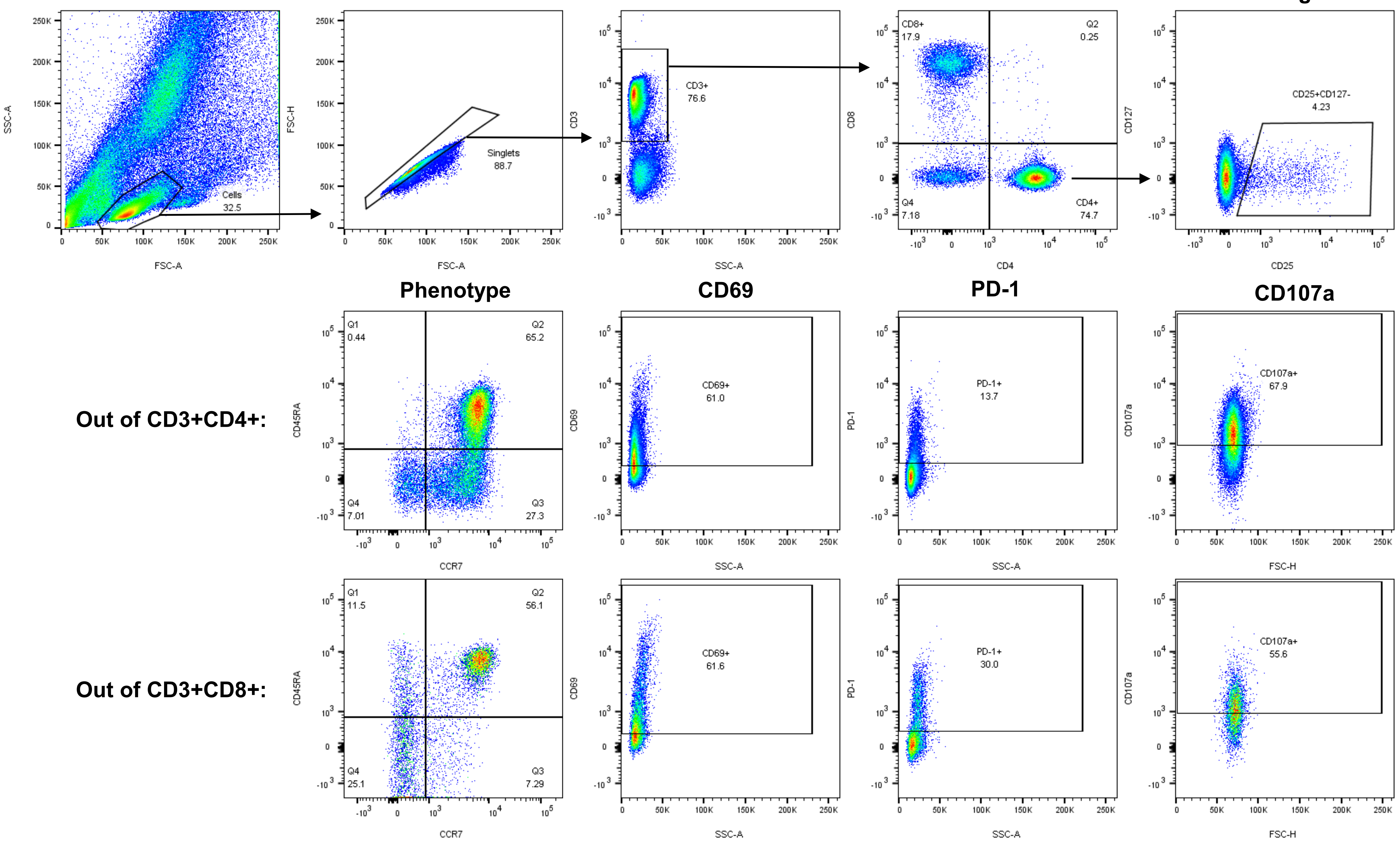

Figure S9: Flow cytometry gating strategy for PBMC/myeloma co-culture. Lymphocytes were gated based on forward (FSC) and sideward scatter (SSC) and singlets were gated from FSC area vs. FSC height. The single cell population was then gated for CD3+ cells, which were separated in CD4+ and CD8+ cells. Both T cell subtypes were gated for the phenotype based on CD45RA and CCR7 expression and for their activation status based on CD69, PD-1 and CD107a expression. Regulatory T cells (CD25+CD127-) were gated on CD4+ cells.
